# The Effect of Emotional Labor of College Administrative Service Workers on Job Attitudes: Mediating Effect of Emotional Labor on Trust and Organizational Commitment

**DOI:** 10.3389/fpsyg.2018.02473

**Published:** 2018-12-14

**Authors:** Sang-Lin Han, Hyeon-Sook Shim, Won Jun Choi

**Affiliations:** ^1^School of Business, Hanyang University, Seoul, South Korea; ^2^Department of Business Administration, Baewha Women’s University, Seoul, South Korea

**Keywords:** job stress, intimacy, professionalism, emotional labor, organizational commitment, trust

## Abstract

Service providers working for a service organization are asked to express such positive emotions as joy, pleasure, and politeness required at the organizational level rather than their natural emotions they are experiencing at the moment. They cannot express their emotion they are actually going through and accordingly, their level of emotional labor and emotional dissonance influence on their job commitment and trust toward their organization. This study thus set out to investigate the effects of leading variables of emotional labor on the level of emotional labor and the impact of emotional labor on organizational trust and organizational commitment with a subject group of college administrative staffs. Three underlying factors such as job stress, intimacy, and professionalism were identified as the determinants of emotional labor. Based on the conceptual background and our research questions, five research hypotheses and the proposed research model regarding the effects of emotional labor on organizational commitment and trust were developed. We also tried to include the moderating effects of work environment and gender of service providers on the research model. Given those findings, this study offers theoretical implication that confirms the negative results of emotional labor. Unlike many different studies on emotional labor from the traditional perspective of service, this study offers a practical implication by expanding and applying it to the field of college administrative service, which is an area where service providers are in the different working environment from the traditional company work environment. Finally, the managerial implications and the limitations of the study were also discussed.

## Introduction

Workers working at service organizations are often required to express positive emotions such as joy, pleasure, and politeness (Sutton and Rafaeli, 1988). In particular, in the case of the service industry, emotional expressions of workers are very important because human contacts are frequently made due to the nature of services. The impressions for a few seconds made through the facial expressions and emotions shown by service workers to customers in the process of service production can be directly connected with the profit of the service company. Nevertheless, there was a lack of interest in emotion studies in the field of service marketing, and interest in emotions in marketing has been mainly shown from the viewpoint of customers. That is, studies have mainly focused on the effects of the emotions experienced by customers in the process of purchasing behavior (Babin and Attaway, 2000; Schaubroeck and Jones, 2000) or how workers should be educated for customer emotion management (Menon and Dubé, 2000; McLaughlin and Carr, 2005). After the studies conducted by Hochschild (1983, 1990), which defined service workers’ efforts to control their actual emotions and express certain emotions that seem to be desirable in the service industry as emotional labor, studies on service workers’ emotions have been actively conducted (Grandey, 2000; Kruml and Geddes, 2000).

Nonetheless, studies on the emotional labor of service workers in the field of administrative services are insufficient. In particular, despite that customers’ evaluation of school administrative services may be more conservative in that the services are delivered to school members, school units’ interest in service workers’ emotion management can be said to be insufficient.

In addition, in the case of administrative services, following the global stream of administrative reform since the 1990s, workers working at service organizations have been required to express mainly those emotions such as joy, pleasure, and politeness (Sutton and Rafaeli, 1988) because the emotional expressions of the service workers positioned at the front lines of service delivery have great effects on the service quality perceived by customers and overall perceptions of the service organizations by the customers. Therefore, in recent years, even public institutions have been regarding emotions as important and have been making effort to break from the existing authoritative forms and derive changes in behavior into the shape of emotional and customer-oriented service suppliers.

However, in the case of service workers who deliver services at the front lines, the situations where they perceive a sort of emotional labor of being unable to express their actual emotions may lead to stress imposed while they work, which may reduce their trust in their organizations or job satisfaction. Eventually, the core of the efforts of workers at educational institutions and administrative service workers can be said to be improving the quality of service and building customer-oriented organization structures and the accompanying work overload may be a leading variable of emotional labor.

As the emotional labor of service providers is regarded as important at service organizations (Adelmann, 1995; Kruml and Geddes, 2000), the present study was intended to investigate the emotion management of service workers in the field of administrative services, which has been studied relatively less extensively thus far (Diefendorff et al., 2005). In particular, the present study was conducted with service workers working at universities with a view to proposing theoretical or practical measures to improve the emotion management of service workers.

The present study aimed to investigate the effects of administrative service workers’ emotion management on the organizational effectiveness perceived at the level of organization dimensions. More concretely, the present study was intended to review variables such as relational effectiveness (job stress, intimacy, and professionalism), emotional labor, and organizational effectiveness (organizational commitment, organizational trust), which are core variables in this study, and examine the causal relations among these variables.

## Conceptual Background and Research Hypotheses

### University Administrative Staff’s Functions and Roles

Although university administrative staff members do not directly engage in educational or research activities, they not only support those activities to ensure that the activities are properly conducted but also play core roles of planning and managing all works in universities. In addition, they not only simply assist professors or students but also are in charge of professional administrative management such as arranging and maintaining all conditions for achievement of the goals of university education. University administrative staff members play the role of a bridge between professors and students while playing pivotal roles in the university administration. Teaching activities are direct instructional activities to achieve the educational goals of school organizations. In contrast, university administration can be said to be service activities intended to support teaching activities and achieve educational goals.

### Emotional Labor

#### The Concept of Emotional Labor

Emotional labor is the concept termed emotional work applied to the situation of job performance in organizations. It can be said to be a concept similar to emotional regulation in that it refers to the effort to intentionally express certain emotions beyond suppressing or controlling certain emotions. However, whereas emotional regulation is individuals’ intentional behavior to change an excessive positive or negative emotional state into the targeted state of emotions expected by themselves or others, emotional labor is more other-oriented and can be said to be a part of emotional regulation appearing in the process of job performance.

The individuals’ efforts to control their actual emotions and express emotions suitable for job performance in organizations as such were conceptualized into emotional labor. In this respect, Hochschild (1983) defined emotion labor as “outward states or facial expressions resulting from induced or suppressed emotions different from actual emotions.”

Based on the argument of Ashforth and Humphrey (1993) and Morris and Feldman (1996) presented leading variables of emotional labor and a multidimensional concept of emotional labor for the results of the variables. Unlike the existing viewpoints, they focus on behaviors to express organizationally desirable emotions with regard to the concept of emotional labor and conceptualized emotional labor based on the diverse dimensions of behaviors to express emotions required by organizations. In this study, the researchers stated that emotional labor consists of four dimensions, which are the frequency of emotional display, the degree of attentiveness required for emotional expressions, the diversity of emotional expressions, and emotional dissonance.

In addition, Grandey (2000) defined emotional labor as a process of expression and regulation of emotions intended to achieve the organizations’ goals. Through this process, rather than focusing on occupational classification, observable expressions of emotions, or the disagreement between the characteristics of the situation and emotions, the internal emotional regulation approach is used more frequently. As such, the concept of emotional labor varies slightly among researchers and no agreed concept is available even now.

#### Emotional Labor in University Administrative Services

Recently, schools and public institutions have been making considerable efforts in activities for customer satisfaction and kindliness to customers such as implementing kindliness education and recruiting kind staff members. These changes can be regarded as indicating that the emotional labor of employees, which had been receiving attention mainly in service positions in the private sector, has become an important form of labor in the public sector too. Although emotional labor began in the form of kind behaviors demanded from administrative service workers in departments related to students mainly organized for civil services in the early days, it appears in all school personnel now along with the transparency, accountability, and information disclosure of individual departments.

School personnel are implicitly required to be receptive, tolerant, understanding, and willing to help so that they can provide services that meet the needs of students while constantly coming into contact with students. Therefore, when they come up with pessimistic feelings, anger, or unkindness about students in the course of delivering administrative services, they make great efforts in emotional regulation in order to suppress such feelings and induce receptive and friendly emotions. The labor for emotional management as such may lead to the feeling of exhaustion as much as does physical work. This psychological aspect as such will greatly affect the intimacy, professionalism, job stress, and organizational commitment of school personnel thereby acting as a factor that affects organizational effectiveness.

#### Previous Studies on Emotional Labor

Hochschild (1983) indicated that emotional labor is directly related to negative behaviors such as drug abuse, alcoholism, and absenteeism and the magnitude of demand for suppression of emotions that must be experienced due to duties was shown to be positively correlated with job stress while being negatively correlated with job satisfaction.

Abraham (1999) conducted a study on customer services (food service industry, telemarketer, apparel retailer, and event company employee) selected from the service industry and indicated that according to the results of the study, job satisfaction (dissatisfaction) mediates the relationship between emotional dissonance and turnover intentions. In addition, job satisfaction, which is affected by emotional dissonance, was shown to be closely related with turnover intentions.

Pugh (2001) studied the process through which the happy emotions expressed by staff members of banks affect the positive emotions of customers, who visit banks, and the positive emotions as such ultimately lead to good perception of the service of banks. Zapf (2002) conducted a study to find what the positive and negative effects of emotional labor on employees who were performing emotional labor were and the results of this study indicated that the positive effect of emotional labor was job satisfaction and the negative effect was emotional exhaustion.

Gountas et al. (2007) examined the effects of flight attendants’ emotional expressions on the emotions of customers as airline passengers. The results of this study indicated that flight attendants’ positive emotional expressions toward customers affected the positive emotions of customers and the positive emotions of customers eventually affected the customers’ overall satisfaction and reuse intentions in relation to the use of the airline.

As such, studies regarding emotional labor have been applied to diverse fields, and discussions on the effects of emotional labor have still produced both positive and negative results. In particular, despite that diverse studies have been conducted in relation to emotional labor, such studies are still insufficient in the field of medical service. The present study was intended to focus on the negative consequences of emotional labor presented by Hochschild (1983).

### Job Stress

Thus far, many scholars have diversely presented concepts related to stress in various fields. They defined stress as an inadequate relationship between environments and individuals and regarded that such an inadequate relationship originates in the desire of humans generated due to the stimulation of environments or regarded the interactions between environments and individuals as being dynamic and the inadequate relationship as a dynamic situation where the solution is perceived to be uncertain in the process of making effort to achieve what individuals desire.

Job stress can be defined as harmful physical or mental responses occurring when what the job demands does not match the employee’s ability, resources, or demands. Job stress occurs in situations where environments and individuals disagree with each other, that is, in situations where the environment requires jobs that exceed the individual’s ability or does not satisfy the individual’s demand.

The first definition of the concept of job stress was made from the viewpoint to see job stress as an external stimulus that affects individuals leading to the destruction of the physical and psychological stability of organization members. From this viewpoint, job stress was defined as “a characteristic of negative environmental factors such as excessive work related to a certain job, role conflicts, role ambiguity, and poor working conditions or job environments that threaten organization members” (Beehr and Newman, 1978). The second definition of job stress was made from the viewpoint of interactions between organisms and environments. From this viewpoint, job stress was defined as “a state where an individual’s ability does not meet the requirements of the job or the job environment provided by the organization does not meet the individual’s desire” (French et al., 1974). Since the subjects of the present study are educational administrative service workers, job stress will be defined as a ‘state of uncomfortable psychological responses felt by employees in the process of performing jobs due to the incongruity among factors related to the job.’ The effects of job stress on the organization are manifested by job dissatisfaction, absenteeism, and turnover, which eventually lead to negative consequences on the organization’s performance.

As for the effects of job stress on psychological tension or emotions, it can be said that as job stress increases, the degree of exhaustion of individuals’ emotions increases too (Meyer et al., 1993). This is also shown by the findings of a study conducted by Sutton (1984) indicating that job stress can ultimately lead to the deterioration of mental and physical health. In particular, if an individual performs emotional labor during which he/she is personally controlled by emotions despite that his/her job stress due to work increases, he/she may perceive emotional labor more intensively because he/she has no personal vent. Therefore, we developed the following hypothesis.

H1: Job stress increases the level of emotional labor.

### Intimacy

“Intimus,” which is the original Latin word for intimacy, means inside or the deepest inside. Therefore, intimacy is related to revealing or sharing one’s deep private parts (Price et al., 1995). The largest asset of an enterprise intimate with its customers is naturally customer loyalty. The characteristics of enterprises that pursue customer intimacy as such are reviewed as follows.

First, such enterprises promote long-term relationships with customers. Enterprises intimate with customers recognize their relationships with customers formed in the long term over time as important investments rather than regarding the profits accrued in the first transaction with new customers as important. Second, such enterprises build and maintain a detailed information system for their customers. Third, such enterprises make effort to continuously provide customers with something more than what customers expect.

The intimacy in the present study can be said to be the degree to which the school staff, who are at the forefront of delivery of services, perceive that they are intimate with students or professors, who are their service customers.

Emotional labor can be regarded as a sort of emotional stress experienced in a situation where it is difficult to express one’s substantive and negative emotions as they are. In cases where service workers perceive intimate emotions toward their service customers highly, they can express their emotions more frankly and this will ultimately make the service workers perceive the degree of emotional labor as being low. Therefore, we developed the following hypothesis.

H2: Intimacy decreases the level of emotional labor.

### Professionalism in Work

The perspective of professionalism in work examined in the present study can be said to be a concept contrary to ‘qualitative work overloading.’ School staff members with professionalism in works that correspond to the areas of individuals were inferred to be highly likely to have high self-esteem for the works under their charge thanks to increases in work efficiency. In addition, since it was inferred through the foregoing that the level of perception of emotional labor by school staff members might be low, the following hypothesis 3 was established.

H3: Professionalism decreases the level of emotional labor.

### Organizational Commitment

Organizational commitment is known to be an important variable explaining the characteristics of organizations as with job satisfaction. Sheldon (1971) regarded organizational commitment as an individual’s positive evaluation of his/her organization or the individual’s will to achieve the organization’s goals. That is, an organization member with strong organizational commitment recognizes the goals of the organization as his/her goals and contributes to the development of the organization.

The reason why diverse studies have been conducted in relation to organizational commitment is that organizational commitment is closely related to the effectiveness of the organizations. Accordingly, many studies have been conducted on the influencing factors that determine organizational commitment. Through a multifaceted study on the leading variables and outcome variables of organizational commitment, Meyer and Allen (1990) presented rewards, the number of years of continuous service, goal identity, positions, age, job satisfaction, job stress, education levels, and job environment as leading variables of organizational commitment and productivity, turnover intentions, absenteeism, and job satisfaction as outcome variables and argued that although the leading variables of organizational are not sufficiently consistent because they are quite diverse, the outcome variables are quite clear.

Eventually, organizational commitment refers to an employee’s intention to devote his/her energy and loyalty to the organization to which he or she belongs. The organizational commitment as such can be defined as a psychological characteristic related to the attitudes of the employees, and it can be said that elements such as loyalty to the organization, the mind to make best efforts for the organization, and self-identification of the employees with the organization are contained in organizational commitment.

It can be inferred that as the intensity of emotional labor increases, the level of attachment or commitment to the organization will became lower. Therefore, the following hypothesis 4 was established.

H4: Emotional labor has negative impact on organizational commitment.

### Organizational Trust

Organizational trust is trust in the objective employment relationship between an organization and its members and it is defined as the ‘members’ overall evaluation and conviction of the organization that the organization will conduct behaviors beneficiary or at least not harmful to the members’ (Zapf, 2002).

Concretely, the reason why trust is necessary in organizations is that trust is important for the development of organizations as follows. First, trust promotes the organization to achieve high performance. Second, trust is an important element in achieving the long-term stability of the organization and happiness of the members. Third, trust induces organization members to have a strong sense of community and voluntarily participate in organizations’ problems thereby enabling organic and human-centered flexible operation of organizations.

Levering (2000) argued that trust is a complex concept consisting of the factors of veracity, respect for individuals, and fairness, and presented three components of organizational trust; first, trust, which is in the relationship between the employees and the management; second, self – esteem, which is in the relationship between each employee and his/her work; and, third, fun, which is in the relationship between the employees who work together.

As such, trust is a very important element of organization’s competitiveness and long-term sustainability in diverse aspects. Therefore, many scholars have promoted studies on interactions between trust and other variables. Such variables include group effectiveness, effective group problem solving, leadership effectiveness, and overall organizational effectiveness.

Trust can be classified by type into emotional trust and rational trust. Lewis and Weigert (1985) conducted a study on trust with a focus on relationships and they classified trust into two axes: emotion and reason. Cognitive trust, which is focused on the aspect of reason, is defined as selecting a person who is trusted based on some good aspects of the person under certain circumstances. This means the formation of subjective trust in relationships with others reflecting aspects that are aimed at pursuing practical interests. On the other hand, emotional trust, which is focused on emotions, is said to be formed through interpersonal emotional ties. That is, trust in others is understood as intimacy and the will to take risks formed through the creation of environments such as emotional ties. In the present study, based on the studies mentioned above, an integrated measurement of trust was made considering both the aspect of emotional trust and the aspect of rational trust.

Service workers who frequently experience emotional labor of being unable to properly deliver actual emotional expressions due to their organizations can be inferred to have low levels of trust in their organizations. Therefore, hypothesis 5 as follows was established.

H5: Emotional labor has negative impact on trust.

### Moderating Effects of Work Environment and Gender

In the present study intended to analyze the effects of emotional labor on university administrative staff members, the effects of emotional labor were regarded to appear differently according to the different work environments of universities or the genders of staff members. We tried to analyze the moderating effects of those two variables. The following research hypotheses were established assuming that the organization culture and working environment of 2 years junior colleges should be different from the research-oriented 4 years universities. Many staff members of research-oriented universities provide services to faculty members and graduate students as well as traditional undergraduate students. However, major role of the staff members of 2 years colleges is focusing on services for students only and in that sense, their working environment could be very different from the each other. Similarly, the perception of working condition and the perceived level of emotional labor might be different depending on gender and, therefore we developed the following hypotheses regarding the effects of moderating variables.

H6: Work environment has moderating effect between the antecedent factors and outcome factors of emotional labor.
H7: Gender of service provider has moderating effect between the antecedent factors and outcome factors of emotional labor.

### Research Model

**FIGURE 1:**
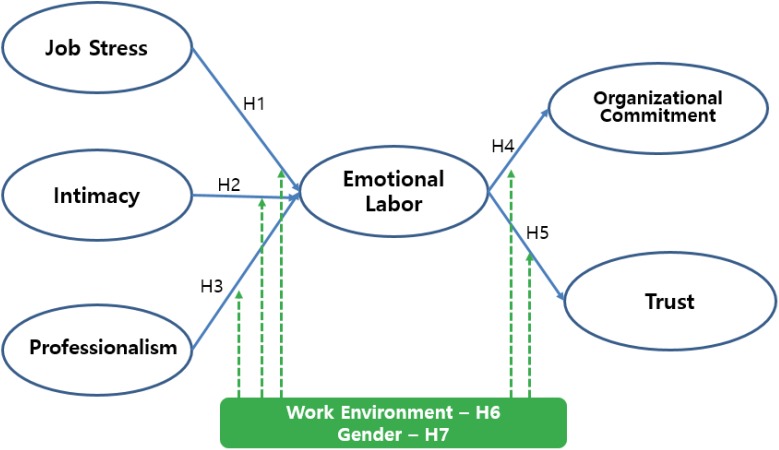
Research model.

Based on the theoretical background, findings of previous studies, and research hypotheses described thus far, we set up a research model as follows (Figure 1).

## Research Methodology and Model Testing

### Questionnaire Survey and Measurement Scale

#### Composition of Job Stress Questionnaire Items

To examine the job stress of administrative service workers, which is the purpose of the present study, the model of Matteson and Ivancevich (1987) was used to make questionnaire items for job stress and, job stress by using Likert 5-point scale. The concrete composition of the questionnaire items for job stress is as follows (Table 1).

**Table 1:** Composition of job stress questionnaire items.

Job stress	Scale items
	(1) I have a lot of work and I am always pressed for time while working.
	(2) My workload has increased significantly.
	(3) The work currently under my charge working on does not seem to fit well with my aptitude.
	(4) My work is not interesting and there is no new change.
	(5) My opinions are respected in many cases.
	(6) I have to work with ambiguous instructions or commands in many cases.
	(7) Sometimes, I have to perform tasks against my judgment.
	(8) I sometimes have to perform tasks without the support of appropriate human and material resources.
	(9) Sometimes, tasks beyond my capability are given to me.
	(10) My role is quite ambiguous.
	(11) My job makes me perform tasks against my values.
	(12) My workplace (school)’s promotion policy ensures fair compensations for tasks performed.


#### Composition of Intimacy Questionnaire Items

In the present study, the six items utilized in a study conducted by Nielsen (2001) were utilized to measure intimacy. The concrete composition of the questionnaire items is as follows (Table 2).

**Table 2:** Composition of intimacy questionnaire items.

Intimacy	Scale items
**Intimacy**	(1) I have a very intimate friend in my team.
	(2) I maintain friendship with my team members even outside the workplace.
	(3) I can tell my team members my secrets.
	(4) I think I can trust my team members.
	(5) I am happy because I can see my team members at work.
	(6) I do not feel anyone in my team as a true friend, no matter with whom I work with.


#### Composition of Questionnaire Items for Professionalism in Work

In the present study, to examine administrative service workers’ professionalism in work, knowledge in related majors, self-improvement, job performance, and commitment to work were examined (Table 3).

**Table 3:** Composition of questionnaire items for professionalism in work.

Scale items for professionalism in work
(1) I have knowledge in majors related with my work.
(2) I have a certificate related with my work.
(3) I am investing my time beyond my daily working hours to handle my work.
(4) I think I have an ability differentiated from other employees for handling my work.
(5) I have a passion for my work.
(6) I think I handle tasks faster than others.
(7) I think I handle my work more accurately than others.


#### Composition of Questionnaire Items for Emotional Labor

Emotional labor is a unique characteristic of service labor. In service labor, “the quality of interactions” between employees and service practitioners *per se* constitutes a part of the service delivered to customers. In the present study, emotional labor was measured using a total of 12 items made based on the study conducted by Morris and Feldman (1996). The detailed questionnaire items is as follows (Table 4).

**Table 4:** Composition of emotional labor questionnaire items.

Scale items for emotional labor
(1) I often smile intentionally rather than truly smiling in my workplace.
(2) I often consciously make effort to make students feel kindness.
(3) I often have to hide my real emotions to lead my work life well.
(4) I feel it is very difficult to express real emotions during my work life.
(5) When I feel bad, I try not to express the emotion.
(6) It is difficult to respond to students with smiles.


#### Composition of Questionnaire Items for Organizational Effectiveness

In the present study, organizational commitment and organizational trust were defined as organizational effectiveness. Organizational commitment can be said to be a strong attitude of service workers to identify the goals of the organization with their goals and try to achieve the goals with positive attachment. The organizational commitment as such was measured with the five items used in a study on organizational commitment conducted by Allen and Meyer (1990). The concept of organizational trust was set as the belief about the organization set by the organization members (Table 5).

**Table 5:** Composition of questionnaire items for organizational commitment and organizational trust.

	Scale items
**Organizational commitment**	(4) I would strongly recommend it if people around me would apply for a job at our school.
	(5) I find joy at my school.
	(6) I like my school more than do other staff members.
	(7) I am not bored when I work at our school.
	(8) I do not consider choosing another school.
**Organizational trust**	(1) My workplace (school) is trying to meet my opinion.
	(2) My workplace (school) tries to treat me fairly.
	(3) I believe that my workplace (school) will make wise decisions for the future of our school.
	(4) My workplace (school)’s field management is streamlined.


### Survey and Data Collection

The subjects of the present study were limited to staff members who provide administrative services at universities and data were collected through questionnaire surveys conducted with the staff members of four universities and two two-year junior colleges located in Seoul, South Korea. For the questionnaire surveys, the researcher firsthand visited the sites to explain the intent of the questionnaire and collected data through one-to-one questionnaire surveys. A total of 250 copies of the questionnaire were collected over approximately 3 weeks and 235 copies excluding questionnaires with unfaithful responses were used in the final analysis.

### Characteristics of Study Respondents

The demographic characteristics of the 235 respondents who responded to the questionnaire in the present study are as follows. As for sex men out numbered women as the percentages of men was 55.3% while that of women was 44.7%. As for ages, the percentage of subjects in their 30 s was the highest at 35.3% followed by those in their 40 s (27.7%), 20 s (24.3%), 50 s (11.5%), and 60 s (1.3%). As for the types of schools where they work, the percentages of universities, two-year junior colleges, and other schools were shown to be 58.3, 40.0, and 1.7%, respectively. As for the number of years of service, the percentage of nine years or more was the highest at 38.7% followed by 1∼3 years (20.0%), less than 1 year (18.3%), 7∼9 years (8.9%), 5∼7 years (7.2%), and 3∼5 years.

### Reliability and Validity of Measurement

Cronbach’s α coefficient was used to verify the reliability of the measurement items used in this study. As shown in Table 6, Cronbach’s α was found to be high, between 0.75 and 0.85. This exceeded the standard (0.70) presented by Nunnally and Bernstein (1994), securing reliability. Confirmatory factor analysis (CFA) was performed on the measurement items to verify the convergent validity of the constructs. The goodness-of-fit index for the CFA was *χ*^2^ = 207.32 (df = 120, *p* = 0.000), CFI = 0.94, and RMSEA = 0.05, satisfying the guidelines from Hu and Bentler (1999). The results of the CFA indicated that the *t*-values of all factor loadings are significant at *p* < 0.001, securing convergent validity. In addition, construct reliability (CR) and average variance extracted (AVE) values that measure internal consistency were examined. The CR value of all variables was 0.70 or higher and the AVE value was 0.5, securing convergent validity.

**Table 6:** Reliability and validity of measurement.

					Cronbach’s
Construct	Scale	Loading^∗^	CR	AVE	alpha
Job stress	JS 1	0.548	0.874	0.777	0.774
	JS 2	0.831			
	JS 3	0.851			
Intimacy	Int 1	0.895	0.933	0.876	0.823
	Int 2	0.818			
	Int 3	0.656			
Professionalism	Pro 1	0.536	0.809	0.680	0.746
	Pro 2	0.860			
	Pro 3	0.746			
Emotional labor	EL 1	0.711	0.922	0.856	0.843
	EL 2	0.867			
	EL 3	0.695			
Organization commitment	Commit 1	0.721	0.930	0.870	0.853
	Commit 2	0.873			
	Commit 3	0.851			
Organization trust	Trust 1	0.540	0.853	0.745	0.758
	Trust 2	0.869			
	Trust 3	0.725			


*^∗^All factor loadings are statistically significant at *p* < 0.001. *χ*^2^ = 207.319; df = 120; *p* = 0.000; TLI = 0.913; CFI = 0.939; RMSEA = 0.049.*

Two methods were used to verify discriminant validity between constructs. First, CFA was performed on each of the items of the constructs included in the research model (Bagozzi and Yi, 1988) to confirm that the correlation between the constructs was less than 1 (Table 7). The results indicated that all correlation values between the constructs were less than 0.85, securing discriminant validity. Second, discriminant validity is secured when the AVE value of the construct is larger than the square value of correlation (AVE > φ^2^), comparing the AVE values of each construct and the square value of the correlation between the two constructs. All standards were met, and discriminant validity was secured.

**Table 7:** Correlation matrix of study constructs^∗^.

	1	2	3	4	5	6
1. Job stress	1.0					
2. Intimacy	0.254	1.0				
3. Professionalism	0.220	0.191	1.0			
4. Emotional labor	0.385	-0.448	0.056	1.0		
5. Commitment	0.060	0.570	0.341	-0.346	1.0	
6. Trust	-0.186	0.317	0.078	-0.370	0.535	1.0


*^∗^All coefficients are significant at *p* = 0.000 level.*

### Verification of the Research Hypotheses and Model Testing

Structural equation model analyses were conducted to verifying the research hypotheses (Table 8). The goodness-of-fit indices of the overall model were shown to be *χ*^2^ = 303.016 (df = 130, *p* = 0.000), CFI = 0.878, TLI = 0.964, RMSEA = 0.067 indicating that the model is a good model that satisfies the criteria of Hu and Bentler (1999).

**Table 8:** Results of hypothesis testing and model analysis.

Path		Standardized estimate	*SE*	C.R	*P*	Result
H1	Job stress → Emotional labor	-0.583	0.097	-7.153	^∗∗∗^	Supported
H2	Intimacy → Emotional labor	0.478	0.148	5.122	^∗∗∗^	Supported
H3	Professionalism → Emotional labor	0.030	0.150	0.464	0.643	Not supported
H4	Emotional labor → Organizational commitment	-0.457	0.073	-4.809	^∗∗∗^	Supported
H5	Emotional labor → Organizational trust	-0.431	0.073	-5.011	^∗∗∗^	Supported


*χ^2^ = 303.016; df = 130; *p* = 0.000; TLI = 0.964; CFI = 0.878; RMSEA = 0.067; ^∗∗∗^*p* < 0.000.*

The results of verification of hypothesis 1 indicated that this hypothesis is statistically significant. The results as such can be said to have confirmed the negative consequences of emotional labor that have been shown in many existing studies and also can be said to provide a practical implication that measures to reduce the job stress of service workers should be prepared at the level of organizations.

The results of verification of hypothesis 2 indicated that this hypothesis is statistically significant. The results as such can be said to be interesting ones suggesting that measures to reduce the variable termed emotional labor should be sought at the level of organizations as well as in terms of personal relationships. These results can be regarded to be very interesting in that whereas many existing studies on emotional labor measured emotional labor mainly from the viewpoint of organizations, the present study suggested that the emotional exchanges of individual service workers are a variable that can reduce emotional labor.

The results of analysis of hypothesis 3 indicated that this hypothesis should not be adopted. In other words, professionalism has no statistically significant effect on the emotional labor. That is, professionalism in work can be regarded to have no statistically significant effect on emotional labor. Although the reduction of work burdens through professionalism in work was predicted to act as a factor to reduce emotional labor in the present study, the results of actual measurement indicated that individuals’ professionalism in work has no effect on emotional labor. Consequently, the reason can be found from the fact that the work of university administrative staff does not require a high level of professionalism. The reason why the professionalism does not influence the level of emotional labor is maybe because the service worker’s job expertise or experience of working knowledge does not necessarily mean the decrease of emotional labor.

The results of verification of hypotheses 4 and 5 indicated that emotional labor has significant effects on both organizational commitment and organizational trust. As mentioned in the research hypotheses, these results can be said to show that emotional labor is an important factor in determining the organizational effectiveness of universities. The above results of verification of the hypotheses were summarized as shown in the following table.

All hypotheses except for hypothesis 3 were shown to have been satisfied. Therefore, on organizing the study findings, it can be seen that job stress is a variable that has significant effects on emotional labor, and that the perception of high levels of job stress lead to the perception of high levels of emotional labor. In addition, it was shown that the perception of intimacy with students leads to the perception of low levels of emotional labor (Hypothesis 2) indicating that organizational support at the level of university is necessary for administrative service workers to form intimate relationships with students. Finally, since emotional labor has significant effects on the variables of organizational effectiveness (organizational commitment and organizational trust), work environment where low level of emotional labor can be perceived should be adapted to improve the organizational performance of administrative service workers of university.

### Analysis of Moderating Effect

#### Moderating Effect of Work Environment

In Hypothesis 6, this study conducted a multi-group analysis to examine the moderating effect of work environment (2- vs. 4-year universities). In order to test the difference between the two different groups, this study analyzed whether there is a *χ*2 difference between the free model and structural weight constrained model to verify the moderating effects.

In Table 9, the *χ*2 difference between the free and constrained models is 280.664, which is much higher than the effective value of *χ*^2^ at the 0.05 significance level. As a result, the hypothesis 6 was supported because the difference between the two models was significant, and the moderating effect of the university work environment was accepted. The moderating effect was verified and the path coefficients between the two groups were compared (Table 10). There is a significant difference between the two groups when the critical ratio (CR) value of the path coefficients of the two groups shows as in the Table 9.

**Table 9:** Comparison of free and constraint models for verification of moderating effect of work environment.

Model	*χ*^2^	df	CFI	RMSEA	Δ*χ*^2^	Δ*χ*^2^/sig.
Free model	1862.229	260	0.842	0.077	-	
Structural weight constrained model	2142.889	295	0.826	0.076	280.664/35	Yes


**Table 10:** Comparison of path coefficients between 2- and 4-year universities.

Path			4-Year	2-Year	CR
Intimacy	→	Emotional labor	-0.685***	-0.531***	4.918
Job stress	→	Emotional labor	0.555***	0.444**	0.541
Professionalism	→	Emotional labor	0.113**	-0.296**	2.330
Emotional labor	→	Organizational trust	-0.604***	-0.647***	1.779
Emotional labor	→	Organizational commitment	-0.550***	-0.757***	1.849


*^∗∗^*p* < 0.01 and ^∗∗∗^*p* < 0.000.*

#### Moderating Effect of Gender

To test the hypothesis 7, we conducted a multi-group analysis to examine the moderating effect of men and women from the perspective of the level of emotional labor. In order to test the difference between the two different groups (men vs. women), this study analyzed whether there is a *χ*2 difference between the free model and structural weight constrained model to verify the moderating effects.

In Table 11, the *χ*2 difference between the free and constrained models is 158.18, which is much higher than the effective value of 55.76 at the 0.05 level when the degree of freedom is 30. As a result, the hypothesis 7 was supported because the difference between the two models was significant, and the moderating effect of gender was accepted. The moderating effect was also verified and the path coefficients between the two groups were compared (Table 12). There is a significant difference between the two groups when the critical ratio (CR) value of the path coefficients of the two groups shows as in the Table 12. Based on this result, we can find that the emotional labor of women is relatively more influenced by the level of intimacy and the emotional labor of men is more influenced by the level of job stress.

**Table 11:** Comparison of free and constraint models for verification of moderating effect of gender.

Model	*χ*^2^	df	CFI	RMSEA	Δ*χ*^2^	Δ*χ*^2^/sig.
Free model	1485.567	260	0.908	0.055	-	
Structural weight constrained model	1643.750	295	0.906	0.052	158.183/35	Yes


*(*χ*^2^-value = 55.76/df of Dif = 30).*

**Table 12:** Comparison of path coefficients between men and women groups.

Path			Men	Women	CR
Intimacy	→	Emotional labor	-0.417***	-0.743***	3.782
Job stress	→	Emotional labor	0.623***	0.363***	2.470
Professionalism	→	Emotional labor	0.009	0.174**	2.033
Emotional labor	→	Organizational trust	-0.407***	-0.551***	1.295
Emotional labor	→	Organizational commitment	-0.463***	-0.353***	1.741


^∗∗^*p* < 0.01 and ^∗∗∗^*p* < 0.000.

## Conclusion

### Discussion and Managerial Implications

Since service workers’ emotion management is regarded as important in the field of services, the present study was intended to be conducted in the aspect of university administrative service workers among the diverse sectors of emotional labor that have been studied thus far. To this end, the present study was intended to be conducted centering on job stress, which is a leading variable of emotional labor that has been presented in many previous studies, with personal variables termed professionalism in work and intimacy, which are aspects where service workers’ individual management is necessary, added to job stress.

In addition, the present study was also intended to be conducted from the perspective about emotional labor believing that organizational effectiveness will be reduced due to the effective emotion management, that is, the management of the norms for emotions expressed by service workers at the level of organization.

To this end, in the present study, the leading variables and outcome variables of emotional labor were extracted from the viewpoint of existing previous studies and for the parsimoniousness of study, it was intended to examine the effects of job stress, professionalism in work, and intimacy, which are leading variables of emotional labor, on the level of emotional labor. Accordingly, interviews were conducted in advance to secure the validity of study subject selection and after securing the validity of study subjects based on the results of the interviews, questionnaire surveys were conducted for empirical analysis with administrative workers working at six universities and school personnel at the points of contacts with students and professors.

The hypotheses were verified based on the results of empirical analysis conducted through a series of processes as such. The results of the analysis are as follows.

First, the job stress of service workers was shown to have increasing (+) effects on emotional labor. It was confirmed that the study findings indicating that the awareness of job stress has significant effects on the emotional labor perceived by service workers in other industries, apply to administrative service workers too. Therefore, it provides a practical implication that conditions under which emotion management can be performed should be prepared at the organization level through the reduction of job stress of service workers.

Second, intimacy was shown to reduce the degree of emotional labor of service workers. This is significant in that the fact that the intimacy perceived by administrative service workers in their relationships with customers affects their personal emotion management was confirmed. In addition, it also provides a practical implication that rather than utilizing only emotion management as a means of control at the level of organization, meetings and events that can form positive relationships with customers (students) should be actively utilized by school organizations to support organization members in relation to their emotional exhaustion with interest.

Third, although the professionalism in work of service workers was thought to have decreasing (-) effects on emotional labor, the results of verification indicated that the decreasing effects were not significant. Based on the foregoing, it can be inferred that the sector of personal abilities does not affect service workers’ emotion management and that individual service workers perceive emotions and their professionalism in work as separate variables.

### Limitations and Future Research Direction

The present study examined the effects of administrative service workers’ job stress, intimacy, and professionalism on their emotional labor, and the effects of emotional labor on organizational commitment and organizational trust in administrative service situations. Therefore, the present study has the implications as mentioned above but it has the following limitations.

First, in-depth discussions on the significance of emotional labor, which is a central concept of discussion in the present study, for job attitudes in administrative services were insufficient. Therefore, qualitative studies are judged to be necessary as future studies because shortcomings may occur since the results of the present study have not been derived from the opinions of all administrative service workers although the opinions were surveyed through in-depth interviews with administrative service workers present in the vicinity.

Second, the present study considered only intimacy, professionalism in work, job stress, and organizational effectiveness in relation to the effects of emotional labor on job attitudes. However, other underlying factors would affect job attitudes in relation to emotional labor. As follow-up studies, it is hoped that multidimensional studies will be conducted on which factors affect emotional labor and job attitude.

Third, although the present study was conducted in the aspect of administrative service workers, in the future, the effects of emotional labor on the performance of students along with the performance of schools should be examined by conducting surveys on students who are provided with the service along with administrative service workers.

## Ethics Statement

This study was carried out in accordance with the recommendations of ethics committee of Hanyang University with written informed consent from all subjects. All subjects gave written informed consent in accordance with the Declaration of Helsinki. The protocol was approved by the ethics committee of Hanyang University.

## Author Contributions

S-LH designed and drafted the work. H-SS designed the work and analyzed the data. WC drafted the work and collected the data.

## Conflict of Interest Statement

The authors declare that the research was conducted in the absence of any commercial or financial relationships that could be construed as a potential conflict of interest.
